# Talk Time Differences Between Interregional and Intraregional Calls to a Crisis Helpline: Statistical Analysis

**DOI:** 10.2196/58162

**Published:** 2024-09-19

**Authors:** Robin Turkington, Courtney Potts, Maurice Mulvenna, Raymond Bond, Siobhán O'Neill, Edel Ennis, Katie Hardcastle, Elizabeth Scowcroft, Ciaran Moore, Louise Hamra

**Affiliations:** 1 School of Computing, Ulster University, Belfast, United Kingdom; 2 School of Psychology, Ulster University, Coleraine, United Kingdom; 3 Samaritans, Ewell, United Kingdom; 4 Samaritans Ireland, Dublin, Ireland

**Keywords:** crisis helplines, call duration, mental health, suicide, suicidal, suicide prevention, population-based, help-seeking behavior, Samaritans, UK, telephony, telephone, telephones, one-way analysis, call, calls, talk time, support, talk time differences

## Abstract

**Background:**

National suicide prevention strategies are general population-based approaches to prevent suicide by promoting help-seeking behaviors and implementing interventions. Crisis helplines are one of the suicide prevention resources available for public use, where individuals experiencing a crisis can talk to a trained volunteer. Samaritans UK operates on a national scale, with a number of branches located within each of the United Kingdom’s 4 countries or regions.

**Objectives:**

The aim of this study was to identify any differences in call duration across the helpline service in order to determine whether service varied interregionally and intraregionally and to determine the impact of calls answered in the same region as the caller, compared with calls answered in a different region on the duration of calls made from landlines to Samaritans UK.

**Methods:**

Calls may be routed by Samaritans, wherein the telephony system sends the call to the next available volunteer, irrespective of location; therefore, individuals may be routed to a branch within the same region as the caller’s current region (intraregional calls) or routed to a branch that is in a different region from that of the caller’s current region (interregional calls). The origin of calls by region was identified using the landline prefix of the anonymized caller identifier, along with the region of the destination branch (as branch details are recorded in the call details record). First, a Levene’s test of homogeneity of variance was carried out for each condition, that is, England calls and Scotland calls. Thereafter, for each condition, a one-way ANOVA or one-way analysis of means was carried out to evaluate any significant differences in call duration.

**Results:**

ANOVA results showed that there are significant differences in call durations between intraregional calls and interregional calls (*P*<.001). Across all conditions within this study, callers stayed on the phone for a shorter period of time when routed to a branch that is within the same region as the call origin than if they were put through to a branch within a different region than the call origin.

**Conclusions:**

Statistical analyses showed that there were significant differences between interregional and intraregional calls. On average, callers to crisis helplines stayed on the phone for a shorter period of time if they were routed to a branch within the same region in which the call originated than if they were routed to a branch in a different region of origin. The findings from this study have practical applications, which may allow crisis helplines to manage their resources more effectively and improve caller satisfaction with the service.

## Introduction

Crisis helplines are one of the oldest suicide prevention resources available for public use [1,2]. They are based on the premise that suicide may be prevented by supporting callers who are in a crisis situation, which is defined as a transient state of psychological disequilibrium where an individual’s coping mechanisms are no longer working [3-5].

In global helpline evidence, characteristics of the call content and call outcomes have been examined [5,6] and helper behavior(s) and intervention styles have been examined through silent monitoring to determine which aspects of helper behavior and intervention styles were significantly related to positive outcomes [7]. In addition, there is evidence of subclasses of callers to crisis helplines, with different call characteristics, such as call duration. These variations may reflect the different needs of callers from each caller group [8-10].

There are many crisis helplines in operation within the United Kingdom; one of the oldest in operation is Samaritans. Samaritans UK is a volunteer-based listening service, where the role of the volunteer is to listen to the caller in a respectful, nonjudgmental, and nondirective manner. The model of crisis helplines such as Samaritans is to offer one-off support; however, many individuals use telephony support more than once and often call the service repeatedly over a period of time [8-10].

Given the role of crisis helplines in suicide prevention, it is important to understand the factors that affect access to these services. National suicide prevention strategies describe the importance of connectedness as a protective factor against suicide [11], which is also a prominent part of theories that explain suicidal behavior [12]; the length of call may reflect the extent to which a caller feels supported and able to discuss his or her personal situation. Depending on predetermined criteria set by the crisis helpline, calls may or may not be routed to the nearest branch to call origin but could be routed to a branch where there is a volunteer available to answer the call. This is common practice in many other telephony-based services, as it allows the client to be connected to a service agent much faster. For example, at Samaritans, callers contacting the service from one region of the United Kingdom may be answered by a branch in a different region of the United Kingdom.

The objective of this study was to assess whether interregional and intraregional calls differ in call duration. Thus, this research seeks to answer the question: Do callers stay on the phone for a longer period of time depending on whether they are put through to a branch within their own region or to a branch within another region?

## Methods

### Data Collection

Data used for this study were provided by Samaritans UK. Calls made to this service originate from the constituent regions within the United Kingdom and are routed to Samaritans branches throughout the United Kingdom. Samaritans operate with a single helpline with no regional routing. This means that callers can be routed to any Samaritans branch within the United Kingdom regardless of where the call originated. The data analyzed in this study were from January 2015 to August 2018. During that period, Samaritans received 25,177,944 calls; of these, 4,647,567 (18.5%) were made from landline numbers. Calls to the Samaritans Welsh language line were excluded from this study. The data contained the following variables: caller ID, an anonymous identifier for the caller; country-specific region of origin (derived from the first number of digits within the caller ID variable representing the prefix for the phone number region); destination branch name (place where call was routed); and call duration. In addition, the average call duration for each caller to each region within the data was computed.

### Ethical Considerations

Ethical approval for this work was provided by Ulster University’s Psychology Filter Committee for application “Understanding Samaritans caller behaviour: a machine learning analysis to identify latent sub population and model caller behaviour,” dated August 8, 2016 (FCPSY-08082016).

### Data Wrangling

This stage consisted of identifying the calls within the data that were made from landline phone numbers. The data contained a “caller ID” variable which is an anonymous identifier for the caller. The first number of digits within the caller ID variable represented the prefix for the phone number region from where the caller made the call. After the end of the prefix in the identifier, the remaining digits are hashed, therefore, anonymizing the caller’s identity. From this, calls that were made by landlines were identified and then extracted for the next stage of the wrangling process.

A landline prefix is specific to an area within a country or region. The calls made by each landline number were categorized by region of origin. A total of 608 places of origin were identified within the data, which equated to 4,647,567 phone calls.

Another variable within the call data that was used was the destination branch name, which is the location of the Samaritans branch where the call was routed. There are 187 destination branches and each of these branches was categorized by region. At this stage, it is now possible to filter calls made from each region of origin to each region of destination. Average call duration for each caller to each region within the data was extracted.

### Statistical Analysis

R Studio (version 3.4.4; Posit PBC) was used to conduct data wrangling and statistical analysis. The ggplot2 package (Posit PBC) [13] was used to create data visualizations. Base R functions (R Core Team) were used to conduct data summaries.

## Results

### Overview

Shorter-duration calls are defined as those less than the mean duration of calls overall, where the mean duration is around 1000 seconds (approximately 16 minutes). Therefore shorter-duration calls are less than 16 minutes, while longer-duration calls are greater than 16 minutes.

The average call duration from callers in each region to other regions was subjected to statistical analysis using Levene’s test of homogeneity of variance and one-way ANOVA or one-way analysis of means. The following subsections detail the analysis for each of the regions in turn, comparing intraregion and then interregion calls.

Throughout the next 4 subsections, the conditions will be referred to as <region of call origin>_<region of branch answering call>. For example, England_NI pertains to calls that originated from landlines in England and answered by a branch in Northern Ireland (NI).

### England to England Versus Other Regions

Levene’s test of homogeneity of variance assumption was violated (*F*_3, 278036_=9.366; *P*<.001). A one-way ANOVA or one-way analysis of means was carried out to uncover any significant differences in average call duration between interregional and intraregional calls. There was a significant difference in average call duration between interregional and intraregional calls for England (*F*_3, 51876_=17.504; *P*<.001).

Table 1 displays the pairwise comparisons of the levels within the England condition. Callers from England stayed on the phone for significantly longer if they were put through to a branch in NI than if they were put through to a branch in England by 38 seconds on average (difference between mean durations = 37.61 seconds; *P*<.001). Callers from England stayed on the phone for longer if they were put through to a branch in Scotland than if they were put through to a branch in England by 31 seconds on average (difference = 31.17 seconds; *P*<.001). Pairwise comparisons of average call duration from England landlines to England branches versus other regions were not significant for all other comparisons (Table 1).

**Figure 1.**
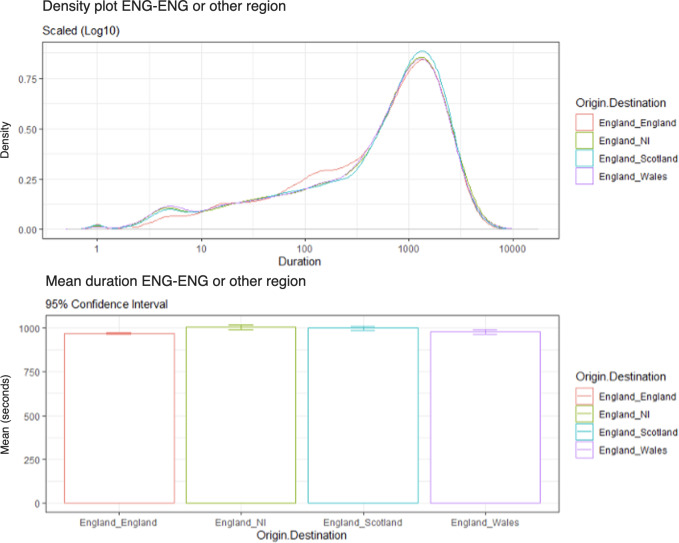
Density plot of call durations from England to England versus other regions (log scale 10) and average call durations (95% CI).

As shown in Figure 1, there was a higher density of calls with a shorter duration made from England to England than in other conditions. Meanwhile, there was a marginally higher density of calls of longer duration made from England to Scotland than in other conditions. Apart from these 2 observations, there was little variation between the conditions elsewhere. Figure 1 also displays the average call duration between England landlines to England branches and other regional branches: ordered from shortest to longest mean duration: England-England (mean call duration 966 seconds), then England-Wales (mean call duration 977 seconds), England-Scotland (mean call duration 997 seconds), and finally England-NI (mean call duration 1003 seconds).

**Table 1.** Pairwise comparisons of average call duration from England landlines to England branches versus other regions.

Group	Difference between means (duration in seconds)	*P* value
England_NI-England_England	37.61	<.001
England_Scotland-England_England	31.17	<.001
England_Wales-England_England	11.52	.13
England-Scotland-England_NI	−6.44	.44
England_Wales-England_NI	−26.09	.01
England_Wales-England-Scotland	−19.65	.04

### Scotland to Scotland Versus Other Regions

Levene’s test of homogeneity of variance assumption was violated (*F*_3, 31477_=15.042; *P*<.001). A one-way ANOVA or one-way analysis of means was carried out to uncover any statistically significant differences between average call durations between interregional and intraregional calls. There was a significant difference in average call duration between interregional and intraregional calls for Scotland (*F*_3, 6528.5_=37.341; *P*<.001).

Table 2 displays pairwise comparisons for each level within the Scotland condition. Callers from Scotland stayed on the phone for 120 seconds longer on average if they were put through to a branch in England than if they were put through to a branch in Scotland (difference = −118.43; *P*<.001). When callers from Scotland were put through to a branch in England, they stayed on the phone for 105 seconds longer than if they were put through to a branch in Wales (difference = −105; *P*<.001). Callers from Scotland stayed on the phone for 120 seconds longer on average if they were put through to a branch in NI than if they were put through to a branch in Scotland (difference = −116.6; *P*<.001). Callers from Scotland stayed on the phone for around 103 seconds longer on average if they were put through to a branch in NI than if they were put through to a branch in Wales (difference = −103.17; *P*<.001). Pairwise comparisons of average call duration from Scotland landlines to Scotland branches versus other regions were not significant for all other comparisons (Table 2).

**Figure 2.**
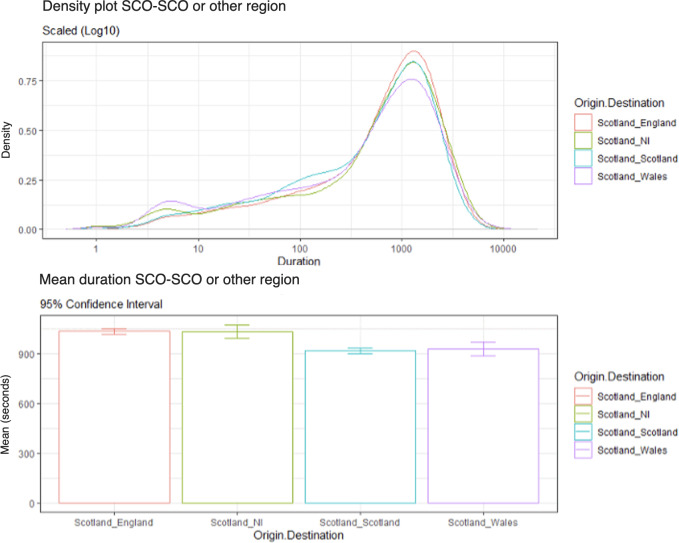
Density plot of call durations from Scotland (SCO) to Scotland versus other regions (log scale 10) and average call durations (95% CI).

As illustrated in Figure 2, a higher density of calls with a shorter duration was observed from Scotland-Scotland than the other conditions. In terms of calls with a longer duration, Scotland-Scotland had the same density as Scotland-NI. Scotland-England had the highest density of calls with a longer duration, while Scotland-Wales had the lowest density of longer duration calls. Figure 2 also displays the average call duration between Scotland-Scotland and other regional branches. Ordered from shortest to longest mean duration: Scotland-Scotland (mean call duration 916 seconds), Scotland-Wales (mean call duration 930 seconds), Scotland to NI (mean call duration 1033 seconds), and finally Scotland-England (mean call duration 1035 seconds).

**Table 2.** Pairwise comparisons of average call duration from Scotland landlines to Scotland branches versus other regions.

Group	Differences between means (duration in seconds)	*P* value
Scotland_NI-Scotland_England	−1.83	.93
Scotland_Scotland-Scotland_England	−118.43	<.001
Scotland_Wales-Scotland_England	−105	<.001
Scotland_Scotland-Scotland-NI	−117	<.001
Scotland_Wales-Scotland_NI	−103.17	<.001
Scotland_Wales-Scotland_Scotland	13.43	.66

### Wales to Wales Versus Other Regions

Levene’s test of homogeneity of variance assumption was violated (*F*_3, 16160_=13.088; *P*<.001). A one-way ANOVA or one-way analysis of means was carried out to uncover any significant differences in average call duration between interregional and intraregional calls. There was a significant difference between interregional and intraregional calls for Wales (*F*_3, 4518_=31.211; *P*<.001).

Table 3 displays pairwise comparisons for each level within the Wales condition. Callers stayed on the phone for 174 seconds longer if they were put through to a branch in England than if they were put through to a branch in Wales (difference [seconds] = −175.23; *P*<.001). Callers stayed on the phone for 168 seconds longer if they were put through to a branch in NI than if they were put through to a branch in Wales (difference [seconds] = −167.93; *P*<.001). Callers stayed on the phone for 138 seconds longer if they were put through to a branch in Scotland than if they were put through to a branch in Wales (difference [seconds] = −137.04; *P*<.001). Pairwise comparisons of average call duration from Wales landlines to Wales branches versus other regions were not significant for all other comparisons (Table 3).

**Figure 3.**
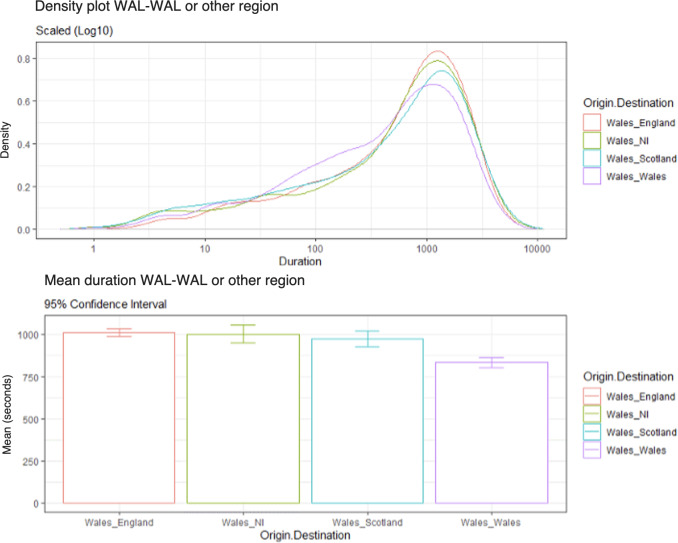
Density plot of call durations from Wales to Wales versus other regions (log scale 10) and average call durations (95% CI).

As shown in Figure 3, there was an increase in variation across all Wales conditions compared with previous regional conditions. There was a higher density of calls with a shorter duration for the Wales-Wales condition. In terms of the calls with longer duration, there was much more variation between conditions. From lowest to highest density for longer duration calls, Wales-Wales had the lowest density of longer duration calls, followed by Wales-Scotland, Wales-NI, and Wales-England. Figure 3 also displays the average call duration between Wales to Wales and other regions; from shortest to longest, Wales-Wales (mean call duration 835 seconds), then Wales-Scotland (mean call duration 972 seconds), Wales-NI (mean call duration 1003 seconds), and finally Wales-England (mean call duration 1010 seconds).

**Table 3.** Pairwise comparisons of average call duration from Wales landlines to Wales branches versus other regions.

Group	Differences between means (duration in seconds)	*P* value
Wales_NI-Wales_England	−7.3	.8
Wales_Scotland-Wales_England	−38.2	.2
Wales_Wales-Wales_England	−175.23	<.001
Wales_Scotland-Wales_NI	−30.89	.46
Wales_Wales-Wales_NI	−167.93	<.001
Wales_Wales-Wales_Scotland	−137.04	<.001

### NI to NI Versus Other Regions

Levene’s test of homogeneity of variance assumption was violated (*F*_3,8626_=8.8469; *P*<.001). A one-way ANOVA or one-way analysis of means was carried out to uncover any significant differences in average call duration between interregional and intraregional calls. There was a significant difference between interregional and intraregional calls for NI (*F*_3,2078_=20.40; *P*<.001).

Table 4 displays pairwise comparisons for each level within the NI condition. Callers stayed on the phone for 143 seconds longer if they were put through to a branch in England than if they were put through to a branch in in NI (difference [seconds] = −142.67; *P*<.001). Callers stayed on the phone for 191 seconds longer if they were put through to a branch in Scotland than if they were put through to a branch in NI (difference [seconds] = 191.47; *P*<.001). Callers stayed on the phone for 176 seconds longer if they were put through to a branch in Wales than if they were put through to a branch in NI (difference [seconds] = 175.74; *P*<.001). Pairwise comparisons of average call durations from Wales landlines to Wales branches versus other regions were not significant for all other comparisons.

**Figure 4.**
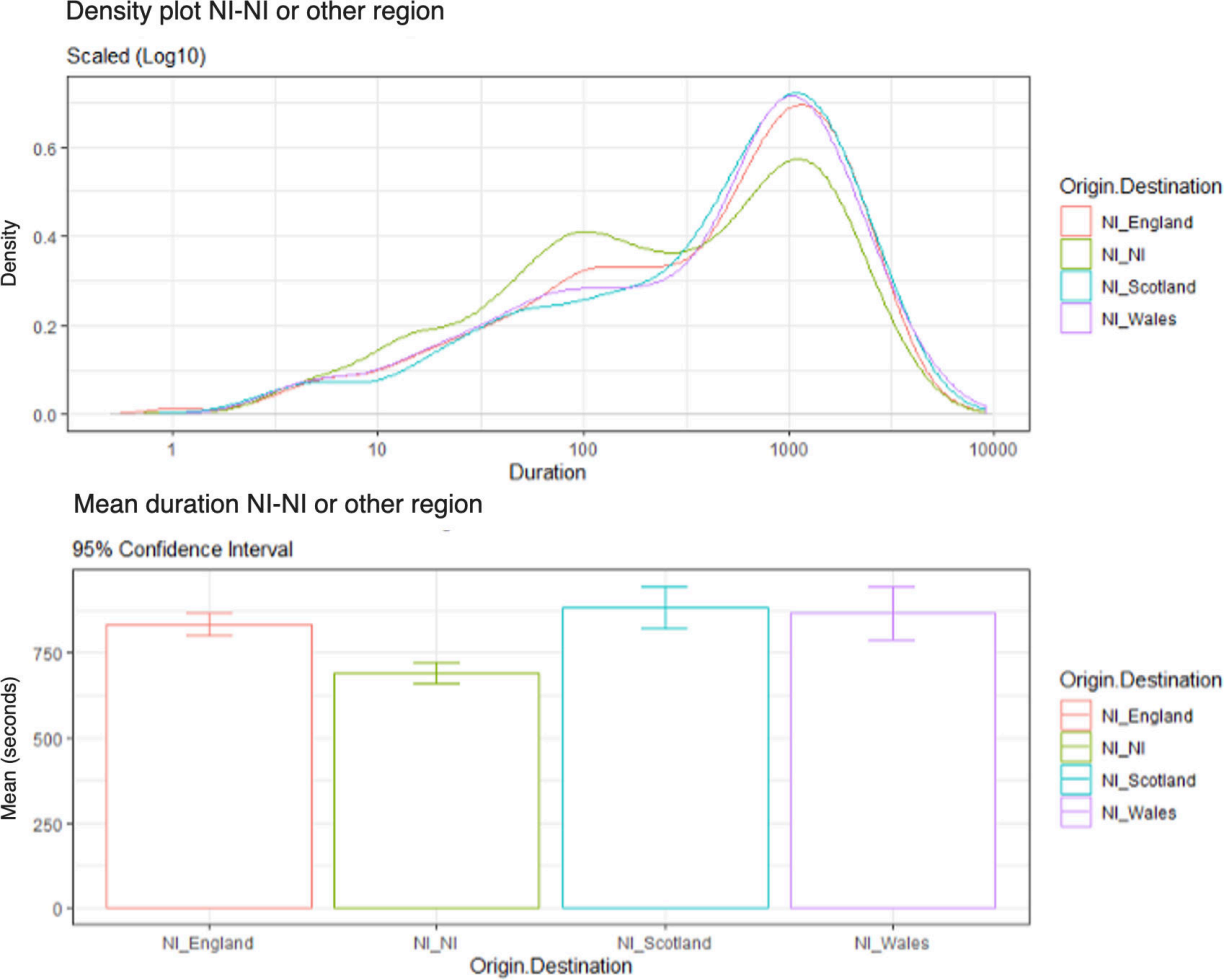
Density plot of call durations from NI to NI versus other regions (log scale 10) and average call durations (95% CI).

Figure 4 shows considerable variation between the NI conditions. There appears to be variation between conditions for shorter-duration calls; NI-NI had a higher density of calls with a shorter duration than the other conditions, followed by NI-England, NI-Wales, and with NI-Scotland having the lowest density of shorter-duration calls. NI-Scotland and NI-Wales had similar density of longer-duration calls, closely followed by NI-England; NI-NI had the lowest density of longer-duration calls. Figure 4 also displays the average talk duration between NI-NI and other regions: from shortest to longest, NI-NI (mean call duration 692 seconds), then NI-England (mean call duration 834 seconds), NI-Wales (mean call duration 867 seconds), and finally NIScotland (mean call duration 883 seconds).

The aim of this study was to explore any potential differences in call duration between interregional calls and intraregional calls within the United Kingdom made to a national crisis helpline. This study analyzed Samaritans UK landline-based calls (N=4,708,205 calls) that were made to the service from January 2015 until August 2018. The origin of the calls was determined based on the landline prefixes that formed the area code digits of an anonymized caller identifier enabling the categorization of calls by region of origin.

**Figure 5.**
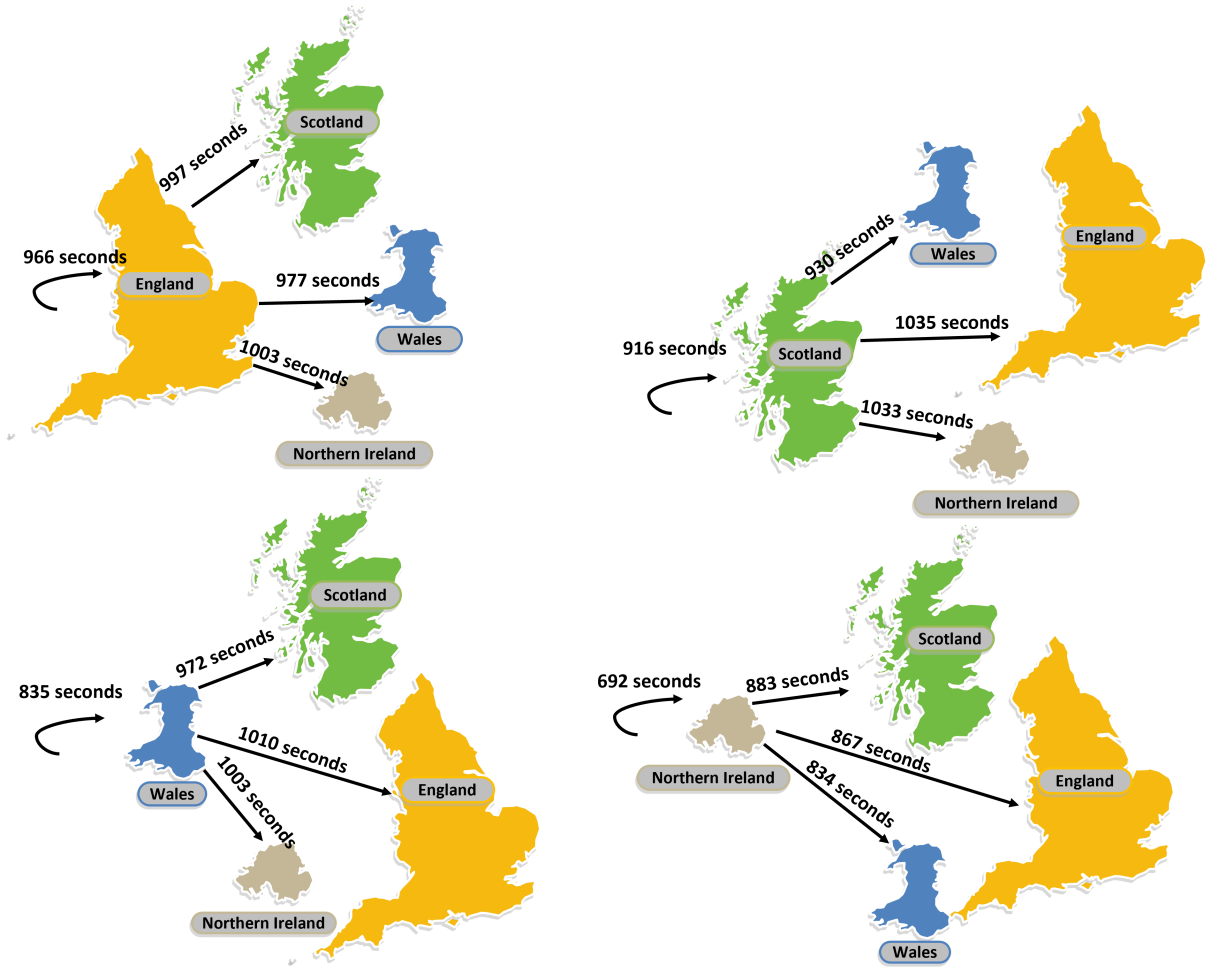
Mean durations of calls between the constituent regions of the United Kingdom.

The destination regions were determined by categorizing the destination branches, which allowed for comparisons of call duration from region to region (Figure 5).

**Table 4.** Pairwise comparisons of average call duration from Northern Ireland (NI) landlines to NI branches versus other regions.

Group	Differences between means (duration in seconds)	*P* value
NI_NI-NI_England	−142.67	<.001
NI_Scotland-NI_England	48.8	.23
NI_Wales-NI_England	33.07	.52
NI_Scotland-NI_NI	191.47	<.001
NI_Wales-NI_NI	175.74	<.001
NI_Wales-NI-Scotland	−15.73	.075

## Discussion

### Principal Findings

Until now, this is an aspect of caller behavior that had not been examined in relation to how callers interact with crisis helplines. Statistical analyses showed that there were significant differences between interregional and intraregional calls. On average, callers to crisis helplines stayed on the phone for a shorter amount of time if they were routed to a branch within the same region in which the call originated than if they were routed to a branch in a different region of origin. Across all regions, there was a higher density of shorter-duration calls for intraregional conditions than in interregional conditions. Conversely, intraregional conditions had the lowest density of longer-duration calls than interregional conditions. Both findings were consistent across all regional conditions.

It is important to point out that the reasons behind different call durations for interregional calls are unknown. It may be the case that callers may feel more encouraged to engage in lengthy conversations and disclose more information about their crisis to a volunteer within a different region due to having a heightened sense of anonymity. While it is the role of the volunteer to have a nonjudgmental approach and uphold confidentiality regarding call information, callers may perceive that there is a risk that they know the volunteer, or that they might have mutual contacts.

Another possible explanation for the significant differences in talk time between regions is that there could be variations in accents within interregional calls. All intraregional call conditions had the shortest mean call duration, had a higher density of shorter-duration calls, and had the lowest density of longer-duration calls. Callers and volunteers may be better able to understand each other if they both speak in the same accent. In contrast, callers and volunteers who are both from different regions may take longer to process the accent of one another. Indeed, studies have examined the impairments of language processing due to foreign accents. Clark and Garrett [14] found that perceptual processing speed of listeners was much slower by an average of 100‐150 milliseconds when sentences produced in the English language were spoken with accented speech (ie, Spanish or Chinese accent) than if spoken in nonaccented speech (ie, native speech). In a series of experiments, Floccia and colleagues [15] concluded that regional accent normalization is exemplified by an initial temporary perturbation in speech processing, resulting in longer reaction times in detecting words spoken in an unfamiliar accent. The authors also state that the differences could also be down to basic differences in stimuli across different accents, meaning that a speaker with a different accent to that of the listener may speak at a various rate. However, this explanation is just one possibility, and this assumes that volunteers have regional accents akin to that area and to date we do not have evidence to confirm that is the case. Another important consideration is the effect of accents more generally, outside of understanding speech or language processing, as it may also relate to how easily the caller identifies with the volunteer and vice versa.

Ultimately, further work should seek to understand the reasons behind differing call durations. For example, future work could include a qualitative analysis with Samaritans volunteers across these regions to explore their views and with callers to explore their thoughts on using the service. Questions for future research include the following: Do callers make a judgment as to a listener’s location (eg, based on accent or local knowledge, or other)? Do callers feel they can identify more with listeners from the same location/with the same accent/who share local knowledge? Does this perception (of location and/or identity) influence willingness to disclose/caller openness/satisfaction with service?

### Limitations

It is not possible to determine the cause of the differences that have been found with any certainty from these data. Some crisis helplines will record qualitative data regarding the content of the call, such as the main presenting reason that was cited by the caller. Additional information such as this may provide important context and some idea as to call complexity. It may be intuitive to think that if a call is complex in nature, that the call will last longer. However, a limitation to this study is that such qualitative information was not available to complement the analysis of the call log data.

It was possible to conduct only the analysis on calls made from landlines within the call log data. In total, while there were 25,177,944 calls within the data set provided by Samaritans UK, only landline calls (18.7% of all calls) were analyzed for this study. Most calls were made from a mobile telephone and it was not possible to determine the location from where these calls were made. Therefore, findings from this study may not be generalizable to all callers who contact crisis helplines as the analysis was limited to landline callers only. In addition, calls in Wales to the Welsh language line were not explored during this study.

### Implications

The findings from this study could have practical applications which may allow crisis helplines to manage their resources more effectively and improve caller satisfaction with the service. In the interests of freeing up service capacity, intraregional routing could potentially allow for more callers to get in contact with a volunteer over time. However, it may be that shorter calls result in less disclosure and thus callers may not prefer this. It may also be the case that callers prefer to speak to a volunteer from the same region, which may promote better understanding by the volunteer of the issues that underpin the caller’s crisis. Qualitative research is warranted to determine caller preferences regarding whether they are routed to a branch within or in a different region from themselves. While results achieved in this study were statistically significant, consideration must be taken by the relevant stakeholders into determining whether these results are, in fact, practically significant. While some statistically significant results yielded a difference upward of 180 seconds between regions, there needs to be careful consideration from crisis helplines who operate a multiregional routing service, as to whether intraregional routing of calls is a cost-effective solution to streamlining the service so that more calls from more callers can be answered at any one time; however, this would have to be balanced against service quality and caller satisfaction.

### Conclusions

This study presents a temporal analysis of more than 4.6 million landline phone calls made to a UK national crisis helpline. The aim was to determine whether callers stayed on the phone for a longer or shorter time depending on whether they were routed to a branch within the same region of origin of their call or in a different region. The study found that across all conditions, callers stayed on the phone for a longer period on average if routed to a branch that was within a different region from which the call originated. Intraregional call conditions had a higher density of shorter calls than interregional call conditions; this finding was also consistent across all conditions. Potential explanations for this finding may be that callers may feel more anonymous if routed to an interregional branch and may disclose more, which leads to longer call times, or it may be the case that callers and volunteers may experience some difficulty in understanding nonnative-accented speech which also results in longer call duration. Another possible reason is that callers find it harder to connect with or identify with the volunteer and thus the calls take longer. While these explanations are speculative, further research is warranted to determine whether intraregional or interregional branches are preferred by callers to national crisis helplines.

## Abbreviations

UK: United Kingdom
NI: Northern Ireland
